# Ultrafast‐Charging Supercapacitors Based on Corn‐Like Titanium Nitride Nanostructures

**DOI:** 10.1002/advs.201500299

**Published:** 2015-10-27

**Authors:** Peihua Yang, Dongliang Chao, Changrong Zhu, Xinhui Xia, Yongqi Zhang, Xingli Wang, Peng Sun, Beng Kang Tay, Ze Xiang Shen, Wenjie Mai, Hong Jin Fan

**Affiliations:** ^1^ Department of Physics and Siyuan LaboratoryJinan UniversityGuangzhouGuangdong510632P.R. China; ^2^ School of Physical and Mathematical SciencesNanyang Technological UniversitySingapore637371Singapore; ^3^ School of Electrical and Electronic EngineeringNanyang Technological UniversitySingapore639798Singapore

**Keywords:** self‐discharge, supercapacitors, titanium nitride, ultrafast charging, ultrahigh capability

## Abstract

**Ultrahigh rates realized by ALD‐made TiN**. The symmetric full‐cell supercapacitors deliver a typical capacitance of 20.7 F cm^−3^ at a scan rate of 1 V s^−1^, and retain 4.3 F cm^−3^ at high rate of 100 V s^−1^. The devices can be charged and discharged for 20 000 cycles with negligible capacitance loss and with an ultralow self‐discharge current (≈1 μA).

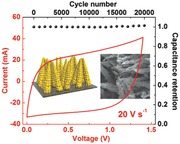

Recent prosperity in multifunctional portable electronic facilities has kindled the strong desire to develop miniaturized energy‐storage devices with high efficiency, long cycle life, and low cost.^1^, ^2^, ^3^, ^4^ Supercapacitor is a promising technology to bridge the gap between batteries and conventional electrostatic capacitors because supercapacitors can demonstrate fast charge and discharge rates, considerable power and energy density, and the ability to endure millions of cycles. Recently, great efforts have been focused on ultrafast‐charging supercapacitors, which can work at charge and discharge rates above 1 V s^−1^.^5^, ^6^, ^7^, ^8^, ^9^, ^10^, ^11^, ^12^ These ultrafast‐charging supercapacitors have either of the following two characteristics in general: one is interdigitated microconfiguration, which can be manufactured using laser writing^6^ and lithography techniques;^5^, ^7^, ^8^, ^13^ the other is the use of carbon‐based electrode material, such as carbon nanoparticles and graphene—other materials have been scarcely reported. Conventional supercapacitors based on noncarbon materials (e.g., metal oxides) face some critical challenges such as low charge rates and severe self‐discharge. Further improvement of the energy and power densities of supercapacitors (especially at ultrafast rates exceeding tens of volts per second) by exploring new electrode materials and/or new nanostructured design with suppressed self‐discharge is urgent.

Metal nitrides have recently attracted increasing attention as suitable electrode materials for high‐performance supercapacitors mainly due to their excellent electrical conductivity. The conductivity of titanium nitride (TiN) varies between 4 × 10^3^ and 5.55 × 10^4^ S cm^−1^,^14^ which is close to that of metals and thus should be beneficial to supercapacitive material that requires fast transport and effective collection of charges.^14^, ^15^, ^16^, ^17^, ^18^, ^19^ Herein, we demonstrate the interesting application of corn‐like TiN nanostructures in ultrafast‐charging energy‐storage devices that break through the limitation of carbon‐based microsupercapacitors. Detailed electrochemical characterization shows that the corn‐like TiN electrodes assembled in a conventional cell device exhibit capacitive performance at scan rates as high as 100 V s^−1^. The resulting ultrafast‐charging TiN supercapacitors show good cyclic stability, ultrahigh power output, and energy storage capacities, which are comparable to existing carbon‐based microsupercapacitors.

**Figure** **1**a,b and Figure S1 (Supporting Information) illustrates the sample structure of the corn‐like TiN on a metal substrate at different fabrication stages. First, Co_2_(OH)_2_CO_3_ nanowire precursor with an average diameter of about 100 nm was obtained by a standard hydrothermal method.^20^, ^21^ Next, the as‐grown nanostructure precursor was coated with a thin TiO_2_ film (20 and 50 nm) by atomic layer deposition (ALD) followed by removal of the cobaltous dihydroxycarbonate precursor in dilute acid. The resulting TiO_2_ nanotubes remain firmly attached to the metal substrate with quasi‐vertical alignment as shown in the scanning electron microscopy (SEM) images. Finally, the corn‐like TiN was obtained by annealing the TiO_2_ in dry ammonia atmosphere. The TiN samples derived from 20 and 50 nm ALD TiO_2_ are named as TiN‐20 and TiN‐50, respectively. The surface of the TiN‐20 nanowires is much rougher than that of TiN‐50 (Figure S2, Supporting Information), which may be because of the limited deformation of thick TiO_2_ shell during annealing in ammonia. The length of corn‐like TiN is about 4 μm as seen in the cross‐sectional SEM image (Figure 1c). High‐resolution transmission electron microscopy (HRTEM) images collected from the particle revealed the lattice fringes of 0.24 and 0.21 nm, which can be assigned to the (111) and (200) planes of the cubic TiN (Figure 1d), respectively. X‐ray diffraction (XRD) patterns (Figure 1d) also support the cubic‐phase TiN. Energy dispersive X‐ray spectroscopy (EDS) mappings trace out the uniform distribution of elements Ti and N (Figure S3, Supporting Information), and no cobalt or carbon remains in the TiN structure. A typical high‐angle annular dark‐field scanning TEM image of TiN and the corresponding line scanning spectra of Ti and N illustrate the hollow interior of the TiN.

**Figure 1:**
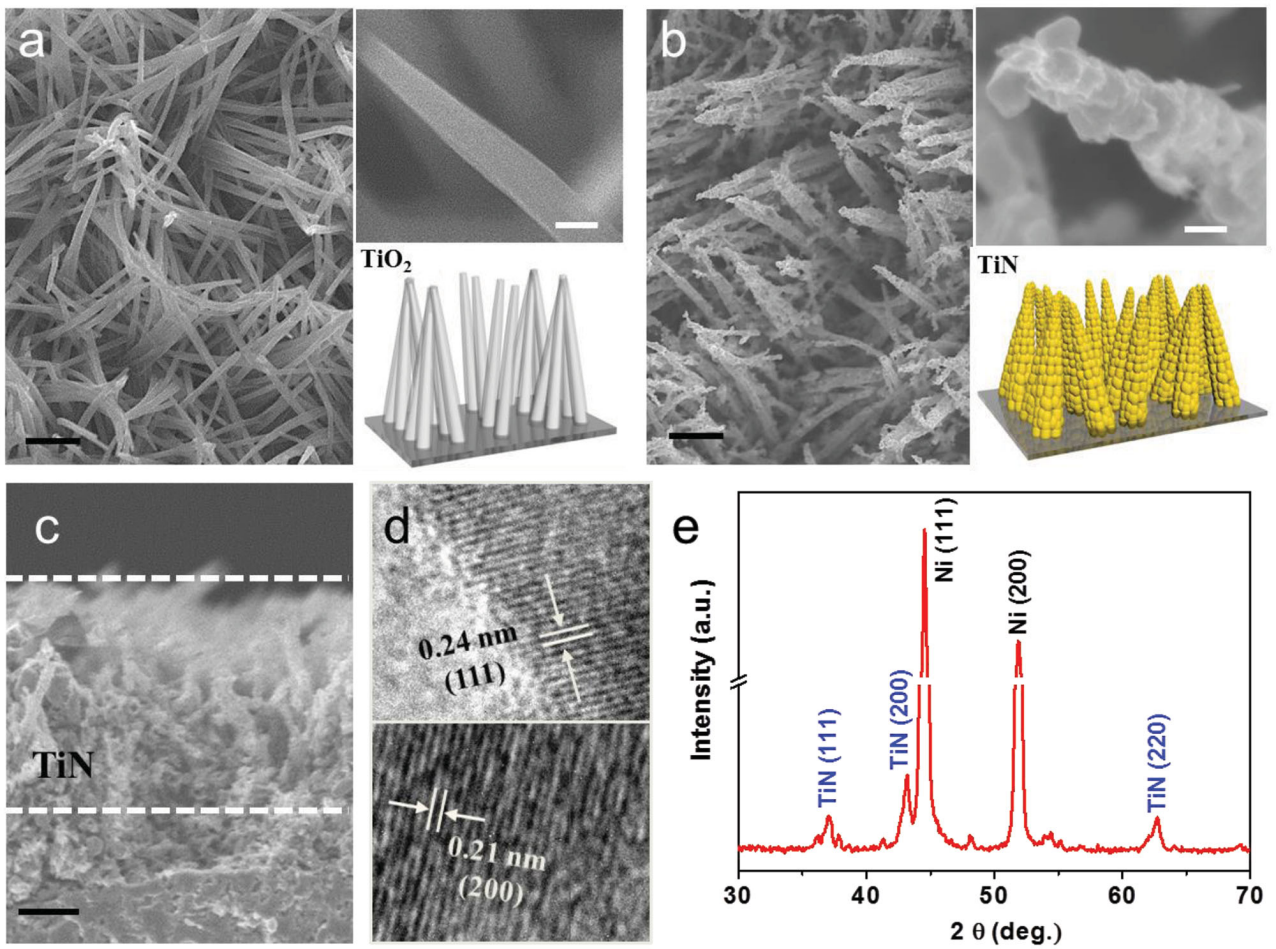
a,b) Schematic illustrations and SEM images showing the TiO_2_ shell and corn‐like TiN‐20. c) Cross‐sectional SEM image of TiN‐50. Scale bar: black 1 μm and white 100 nm. d) TEM images of TiN‐20. e) XRD patterns of TiN on Ni plate. The three diffraction peaks at 36.7°, 42.6°, and 62.2° correspond to the cubic phase TiN (PDF 38‐1420).

To evaluate the power capability of the ultrafast TiN‐based supercapacitors, cyclic voltammetry (CV) experiments were performed at scan rates ranging from 0.1 to 100 V s^−1^ using a symmetric capacitor configuration with organic electrolyte (**Figure** **2**; Figure S4, Supporting Information). The rectangular CV curves at high scan rates indicate a nearly ideal electrical double‐layer capacitive behavior, since pseudocapacitive materials usually offer energy and power under a discharge time domain from 10 s to 10 min.^22^ The TiN nanoparticles on each corn offer enlarged surfaces that are easily accessible for ion adsorption. Figure S5 (Supporting Information) illustrates schematically the charge and discharge process of the TiN electrode. When a fast charge action is applied, the negative ions will be quickly absorbed onto the positive TiN particles; when the scan polarity is reversed, the attracted ions will instantly return to the electrolyte. On the contrary, the polarity of the adions on negative electrode is positive during charge. Surface functional groups are confirmed existing on the surface of TiN nanostructures by X‐ray photoelectron spectroscopy (XPS) measurement (**Figure** **3**). Based on previous report of metal nitride,^23^ the surface functional groups (oxides) formed on the surface of metal nitride are also believed to help the fast energy storage process by redox reaction process (as a supplementary for electrical double‐layer capacitor). In addition, it has been reported that titanium oxynitride thin films with low oxygen contents are partly metallic suggesting that the oxynitride layer on the surface of TiN nanoparticles may have relatively higher electrical conductivity.^24^ The linear dependence of discharge current on the sweep rate was observed at scan rates up to 20 V s^−1^ as shown in Figure 2f, because of the optimization of the hierarchical tube and particle structures.

**Figure 2:**
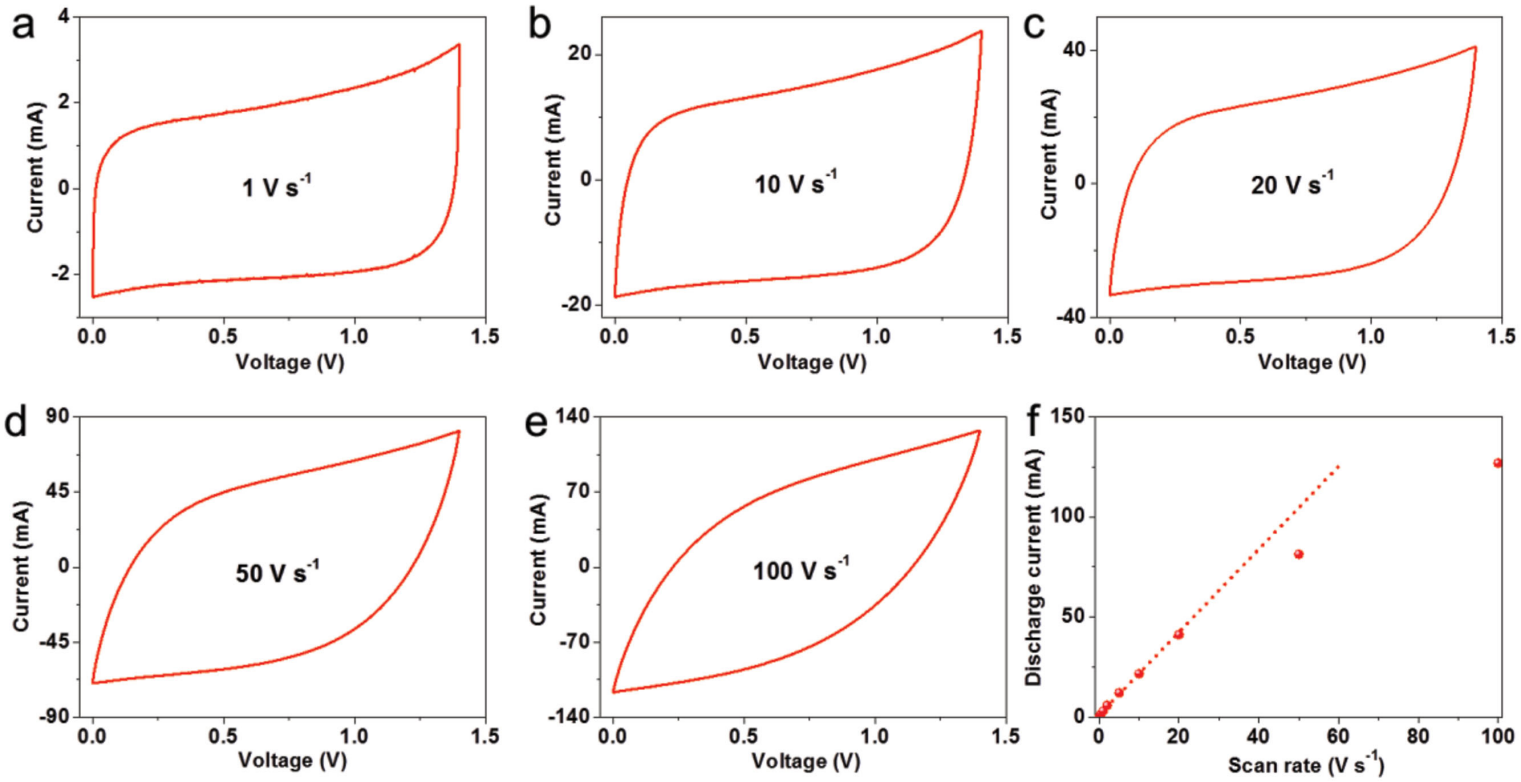
Electrochemical characterizations of the TiN‐20 device. a–e) CV curves obtained at different scan rates in a 1 mol L^−1^ LiClO_4_/anhydrous acetonitrile. A typical rectangular shape, as expected for double‐layer capacitive materials, is observed at ultrahigh scan rates. f) Evolution of the discharge current versus scan rate. A linear dependence is obtained at scan rates up to 20 V s^−1^ in the capacitive region, indicating ultrahigh power ability for TiN devices.

**Figure 3:**
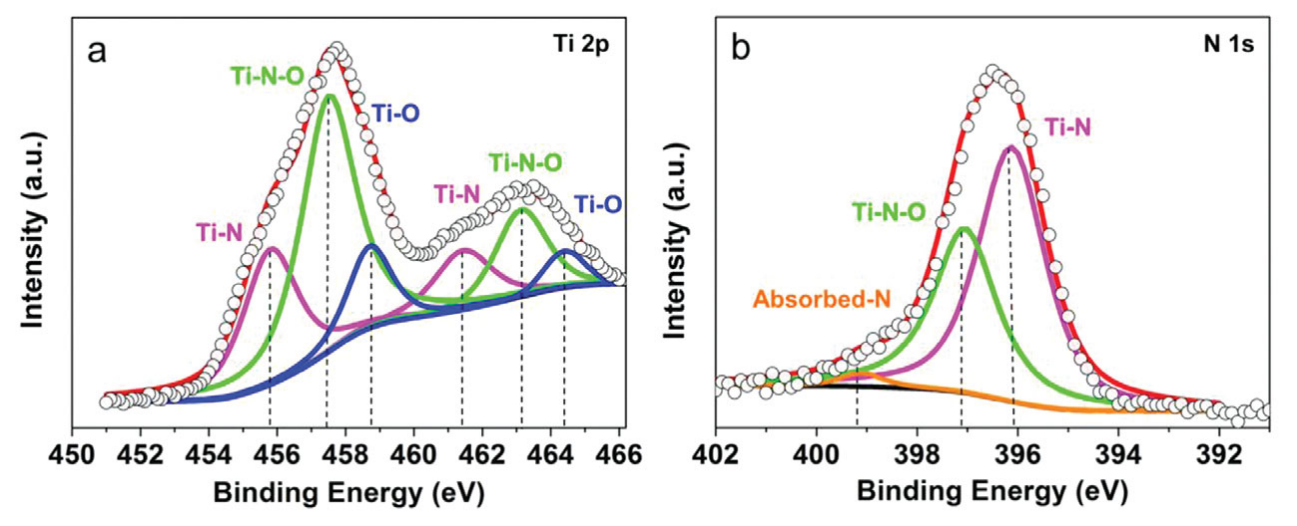
Surface analysis of the TiN nanocorns. a) Core‐level Ti 2p XPS spectra and b) core‐level N 1s spectra collected for the TiN electrode.

The surface of the TiN particles is highly accessible to the electrolyte, with very little impediment to ion transport, providing high capacitance even when operated at ultrahigh charge and discharge rates. **Figure** **4**a,b shows a comparison of the volumetric capacitance and energy density of the device. At a scan rate of 1 V s^−1^, TiN‐50 and TiN‐20 exhibit a volumetric capacitance of 20.7 and 10.0 F cm^−3^, respectively, higher than that of active carbon (AC; 9.0 F cm^−3^) and onion‐like carbon (1.3 F cm^−3^) under the same measurement condition.^8^ Note that data of the volumetric performance in previous literature are often not provided for conventional supercapacitors, causing difficulty in direct comparison. As shown in Figure 4a, the capacitances of TiN‐50 nearly double that of TiN‐20 below the scan rate of 20 V s^−1^ due to the higher mass loading and denser nanostructure in the former. However, the TiN‐20 shows better capacitance retention (i.e., rate capability) than the TiN‐50 when increasing the scan rate from 0.5 to 100 V s^−1^ (see Figure S6 in the Supporting Information). This may indicate a more facile ion transport for the TiN‐20 electrode.

**Figure 4:**
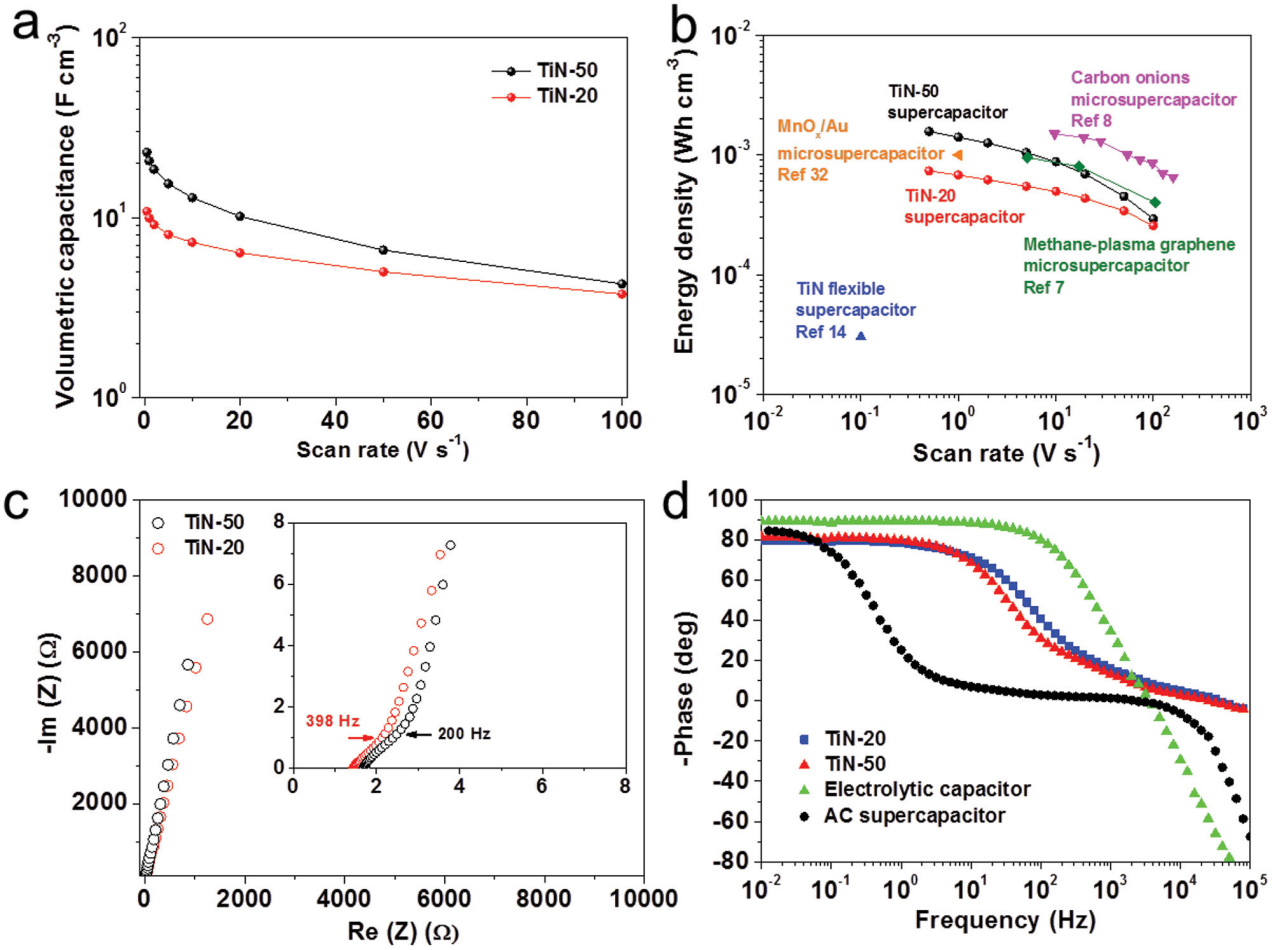
Comparison of TiN supercapacitors and other energy‐storage devices. a) Evolution of the volumetric capacitance versus scan rate. b) Volumetric energy density as a function of scan rate. Data from other energy‐storage devices are included for comparison. c) Impedance spectra of TiN devices. Inset shows the zoom‐in view of the impedance spectra in the high frequency region. d) Phase angle versus frequency of the TiN supercapacitors in comparison with the commercial electrolytic capacitor and AC supercapacitor.

To gain further insight into the mechanism of the ultrafast characteristic upon electron/ion transfer of TiN, the electrochemical impedance spectroscopy (EIS) was measured. The Nyquist plot shown in Figure 4c demonstrates capacitive behavior even at high frequencies (398 Hz for TiN‐20 and 200 Hz for TiN‐50) owing to the highly accessible surface of the corn‐like TiN. The equivalent series resistance obtained from the intercept of the plot on the real axis is only 1.4 Ω for TiN‐20, manifesting its good electric conductivity and low internal resistance of TiN electrode. The frequency at −45° marks the point at which the resistive and capacitive impedances are equal; this is also known as the characteristic frequency of one device.^25^ The characteristic time constant (*τ*_characteristic_) is the time required to discharge the device with an efficiency of 63.2% and can be calculated by 1/(2*πf*_characteristic_).^6^, ^26^ The *τ*_characteristic_ values of TiN‐50 and TiN‐20 device are calculated to be 4 and 2 ms (the corresponding characteristic freqencies are 40 and 80 Hz, respectively), in comparison with 400 ms for the AC supercapacitor and 0.3 ms for the electrolytic capacitor (Figure 4d). These extremely small *τ*_characteristic_ values for the corn‐like TiN are very promising compared with previously reported values for ultrafast‐charging supercapacitors: onion‐like carbon (26 ms),^8^ poly(diallyldimethylammonium chloride)‐graphene (33 ms), and laser‐scribed graphene (19 ms).^6^ It should be noticed that a 2*π* factor was not included in previous work according to different definition of the time constant.^26^, ^27^ These results strongly suggest that our TiN possesses great potential for instantaneous delivery of ultrahigh power and energy.

A comparison of the different energy‐storage devices designed for high‐power device applications is presented in the Ragone plots (Figure S7, Supporting Information). The data of TiN supercapacitors, as well as a commercial high‐energy lithium thin‐film battery (4 V/500 mAh) and high‐power aluminum electrolytic capacitor (3 V/300 mF) are included.^8^, ^10^ Remarkably, our TiN supercapacitors deliver a volumetric energy density of 1.5 mWh cm^−3^, which is comparable to the previous carbon‐based microsupercapacitors and lithium thin‐film batteries (10^−3^−10^−2^ Wh cm^−3^), and much higher than the previously demonstrated flexible TiN supercapacitor device (0.05 mWh cm^−3^).^14^ The very high power density of our TiN supercapacitors, 150 W cm^−3^ at an energy density of 0.30 mWh cm^−3^, implies the capability of discharging within an extremely short time (14 ms). Detailed comparisons are listed in Table S1 (Supporting Information).^5^, ^6^, ^7^, ^8^, ^28^, ^29^, ^30^, ^31^, ^32^ To our best knowledge, this is the first report of noncarbon supercapacitors without microconfiguration having such excellent performance in terms of ultrahigh power and energy densities.

Stability and reliability of the supercapacitor devices are evaluated. These TiN devices show extremely stable cycling performance, retaining nearly full initial capacitance after 20 000 charge–discharge cycles. From the inset CV curves (at a scan rate of 10 V s^−1^), the capacitance of TiN‐20 supercapacitor increases by about 5% after 20 000 cycles (**Figure** **5**a). The slight increase of capacitance may be related to the surface activation progress during the cycling.

**Figure 5:**
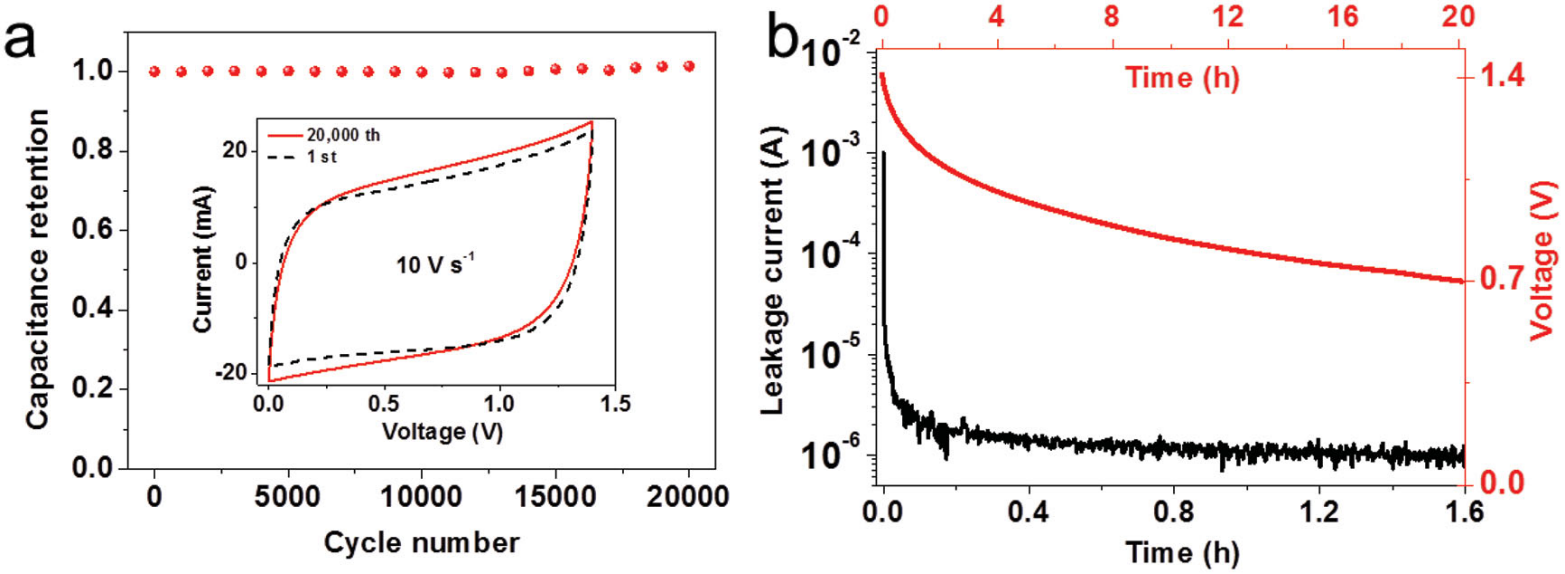
a) Capacitance retention of the TiN‐20 device. The capacitance shows a flat profile with a slight increase by about 5% after 20 000 cycles. b) Leakage current and self‐discharge characteristics of the TiN‐20 supercapacitor.

Other significant factors in practical applications are leakage current and self‐discharge of the device.^1^ These issues have received very limited concern in the fast‐growing literature of supercapacitive devices. During self‐discharge, a small leakage current will cause voltage decay of a charged supercapacitor over time. The leakage current can be measured by applying a rated DC voltage (usually *U*_max_) to the device and monitoring the current while retaining the voltage. It is found from Figure 5b that the leakage current dropped significantly after 10 min (from 1 mA to 1 μA). Such a low leakage current is highly desirable because it implies insignificant shuttle reactions caused by impurities in the electrode. With the small leakage current, our TiN device may be integrated with energy harvesters to create efficient self‐powered systems.

The self‐discharge curves can be obtained immediately after charging the device to *U*_max_. Basically, the voltage difference between the two terminals of the supercapacitor is recorded on an open circuit as a function of time. In general, most supercapacitors are operated in the range of *U*_max_ to 1/2 *U*_max_, so the time required for the voltage across the supercapacitor changing from *U*_max_ to 1/2 *U*_max_ was measured. Figure 5b shows that our TiN device retains an output voltage of 0.7 V after 20 h. This value is better than that of the laser‐scribed graphene microsupercapacitor (13 h) and close to that of the commercial supercapacitors (21 h).^6^ The advantages of low self‐discharge track and high power output are very desirable for the applications of the devices in areas of standby power or fast‐reaction charging electronics. In this work, the improved conductivity and the array structure of the TiN nanocorns are believed to play important roles. The conductivity of TiN is much higher than those of the common oxide pseudocapacitive materials.^33^, ^34^, ^35^ The array structure with open gaps also helps to facilitate the electrolyte ion diffusion. Similar reasons have been adopted to explain the outstanding supercapacitor performance in 2D graphene and Mxene.^6^, ^36^

In consideration of practical use, combination of the supercapacitor devices is demonstrated for the purpose of increasing the operating voltage or output current. **Figure** **6** shows the CV curves and discharge profiles of three TiN‐20 supercapacitors in both series and parallel configurations (packaged in alligator clips). The tandem devices exhibit essentially rectangular CV curves and triangular charge–discharge profiles. Importantly, the increase of voltage and currents in series and parallel combination, respectively, are in full accordance with the capacitor characteristics. It is expected that the ultrafast‐charging TiN supercapacitors can power a wide range of electronic devices. To confirm the universality of the electrochemical performance of TiN, TiN‐20 structures were synthesized on carbon fabric for ultrafast‐charging flexible supercapacitor applications. Figure S9 (Supporting Information) shows the electrochemical performance of TiN‐20 on carbon fabric. The morphology of TiN‐20 on carbon fabric is denser than that of the sample grown on metal substrate. The assembled device shows nearly rectangular CV curves at high scan rates, indicating good capacitive behavior. The characteristic time constant of carbon fabric based device is as small as 2.5 ms, approximating to the value on metal substrate device because of the similar construction of materials. The demonstration of TiN‐20 grown on carbon fabric presents the potential of ultrafast‐charging supercapacitors in flexible and wearable electronics.

**Figure 6:**
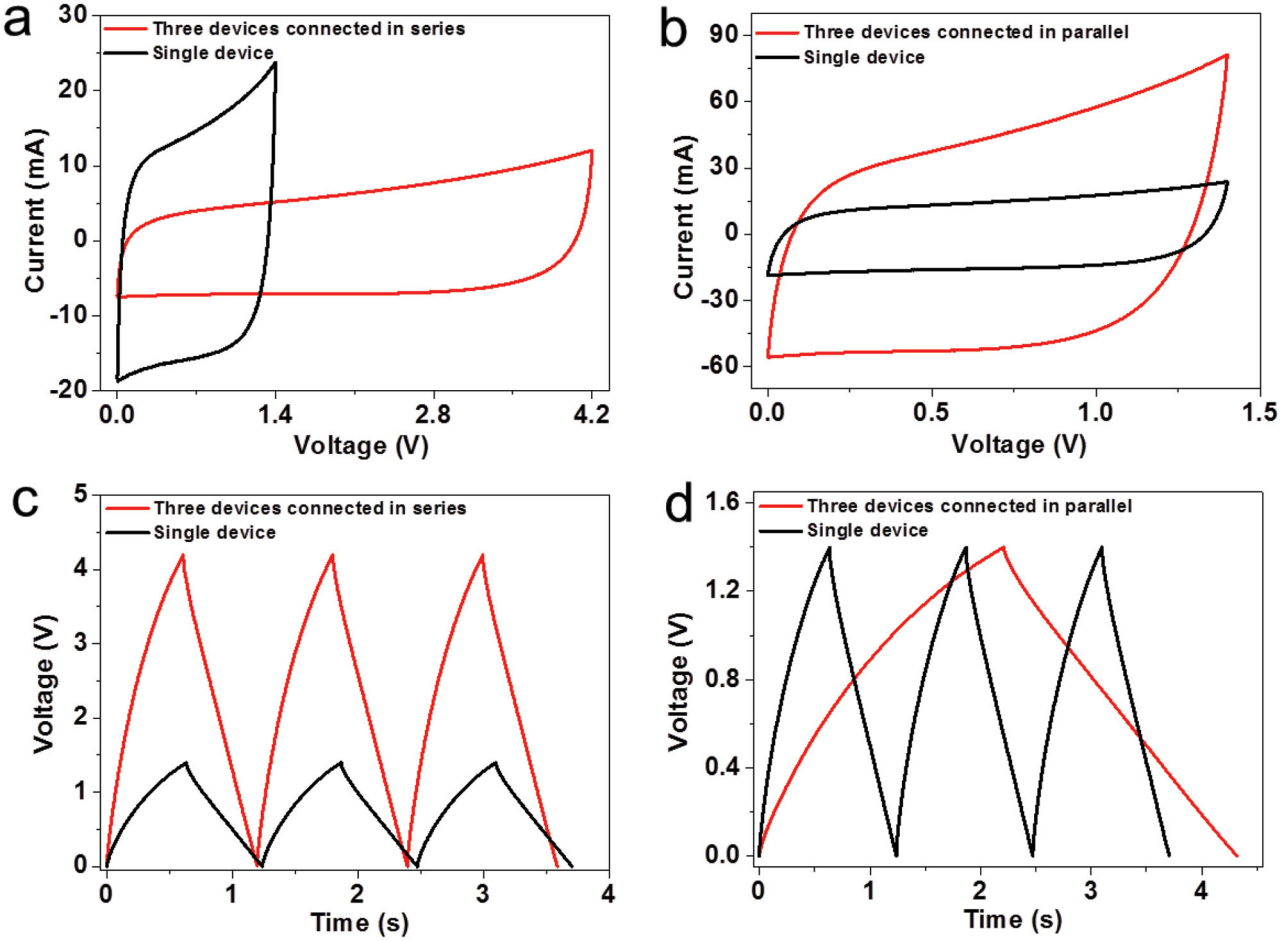
Electrochemical performance of three TiN‐20 supercapacitors connected in a,c) series and b,d) parallel. The scan rate in (a,b) is 10 V s^−1^. The charge and discharge current in (c,d) is 5 mA.

In summary, we have demonstrated a prototype of TiN‐based supercapacitors that exhibit ultrafast charging capability and slow self‐discharge without microconfiguration. The fabrication of the TiN electrodes is realized by ALD of TiO_2_ on nanowire precursor followed by ammonia annealing. Such devices deliver high volumetric capacity, high energy and power densities, and negligible capacity loss after 20 000 cycles. A low leakage current (around 1 μA) and slow self‐discharge, two technically important features, are also obtained. We have also demonstrated series and parallel combinations of the TiN‐based supercapacitors. These ultrafast‐charging TiN‐based supercapacitors can directly power high‐power microelectronics, and their application may also be extended to flexible and wearable electronics.

## Experimental Section

*Material Synthesis*: Corn‐like TiN nanostructures grown on different substrates (nickel plate, stainless steel, or carbon fabric) were prepared by the following process. Firstly, self‐supported Co_2_(OH)_2_CO_3_ nanorod arrays were prepared by a hydrothermal synthesis method following previous reports.^20^, ^21^ The solution was prepared by dissolving 2 mmol Co(NO_3_)_2_·6H_2_O, 4 mmol NH_4_F, and 10 mmol CO(NH_2_)_2_ in 60 mL distilled water. The resulting solution together with the substrates was transferred into Teflon‐lined stainless steel autoclave. The sealed autoclave was heated in an electric oven at 120 °C for 4 h, and then allowed to cool down to room temperature. The samples were then washed with distilled water and dried in furnace at 90 °C. For the second step, the samples were coated with a layer of 20 or 50 nm TiO_2_ by ALD (Beneq TFS200) using TiCl_4_ and water as the titanium and oxygen source, respectively. The deposited temperature was set at 120 °C. After the TiO_2_ shell coating, the samples were immersed in 1 mol L^−1^ HCl aqueous solution for 20 min to remove the Co_2_(OH)_2_CO_3_ template. Finally, the TiO_2_ shells were converted to corn‐like TiN by annealing in ammonia at 800 °C for 1 h. The mass loading of TiN was determined ≈1.01 and 2.30 mg cm^−2^ for TiN‐20 and TiN‐50, respectively.

*Supercapacitor Device Fabrication*: The electrochemical performance of the corn‐like TiN was tested in CR2032 coin‐type symmetric supercapacitors, which were fabricated in atmosphere. The electrolyte was an organic electrolyte with 1 mol L^−1^ LiClO_4_ in nonaqueous acetonitrile solution. The positive and negative electrodes (ca. 1 cm^2^ in diameter) were separated by a porous nonwoven cloth separator and assembled into coin‐type symmetric cells.

*Characterization and Measurements*: The microstructure and phase of samples were characterized by SEM (FEI SIRION), HRTEM (JEOL JEM‐2100F), XPS (ESCALab250, Thermo VG), and XRD (Rigaku, MiniFlex600, Cu Kα). The electrochemical performance was investigated by CV, galvanostatic charge/discharge, and EIS measurements. CV and charge/discharge testing were performed on a CHI660C electrochemical workstation (Chenhua, Shanghai). EIS measurements were recorded on a VersaSTAT3 electrochemical workstation (Princeton Applied Research, USA) over a frequency range of 100 kHz to 10 mHz with an amplitude of 10 mV at the open‐circuit potential. All experiments were performed at room temperature.

*Calculations*: The volumetric capacitance (in F cm^−3^) was calculated using the voltammetric discharge integrated from the cyclic voltammogram over the whole potential range (1.4 V), according to the following equation

(1) $$C=\frac{Q}{UV}$$

(2) $$Q\;=\;\frac{\oint IdU}{2v}$$

where *Q* (*C*) is the average charge, *U* (*V*) is the potential window, *v* is scan rate (V s^−1^), and *V* (cm^3^) is the synthesized TiN layer volume in the micrometer range.

Energy density (*E*, Wh cm^−3^) and power density (*P*, W cm^−3^) are calculated as

(3) $$E\;=\;\frac{1}{2}CU^{2}$$

(4) $$P\;=\;\frac{E}{\Delta t}$$

where Δ*t* is the discharge time and *C* is volumetric capacitance calculated before.

The time constants can be calculated by

(5) $$\tau_{\mathrm{frequency}}\;=\;RC\;=\;Z^{\prime }\left(-\frac{1}{2\pi fZ^{\prime \prime }}\right)$$

where *Z*′ is the resistive element, *Z*″ is the capacitive element, and *f* is the frequency.^26^

The characteristic time constant, *τ*_characteristic_, which is taken at the −45° phase angle where the real and the imaginary component have equal magnitude (*Z*′ = −*Z*″), is calculated using

(6) $$\tau_{\mathrm{characteristic}}\;=\;\frac{1}{2\pi f}.$$

## Supporting information

As a service to our authors and readers, this journal provides supporting information supplied by the authors. Such materials are peer reviewed and may be re‐organized for online delivery, but are not copy‐edited or typeset. Technical support issues arising from supporting information (other than missing files) should be addressed to the authors.

Supplementary
