# Supplementary material for: Ultrafast‐Charging Supercapacitors Based on Corn‐Like Titanium Nitride Nanostructures
Source: Adv Sci (Weinh). 2015 Oct 27;3(6):1500299. doi: 10.1002/advs.201500299 (PMC5057334; doi:10.1002/advs.201500299)
Supplement: Supplementary file 1 — Supplementary [file ADVS-3-0l-s001.pdf]

Copyright WILEY-VCH Verlag GmbH & Co. KGaA, 69469 Weinheim, Germany, 2015.

Supporting Information

for *Adv. Sci.*, DOI: 10.1002/advs.201500299

Ultrafast-Charging Supercapacitors Based on Corn-Like Titanium Nitride Nanostructures

Peihua Yang, Dongliang Chao, Changrong Zhu, Xinhui Xia, Yongqi Zhang, Xingli Wang, Peng Sun, Beng Kang Tay, Ze Xiang Shen, Wenjie Mai, and Hong Jin Fan**

Copyright WILEY-VCH Verlag GmbH & Co. KGaA, 69469 Weinheim, Germany, 2013.

Supporting Information

Ultrafast-Charging Supercapacitors Based on Corn-Like Titanium Nitride Nanostructures

Peihua Yang, Dongliang Chao, Changrong Zhu, Xinhui Xia, Yongqi Zhang, Xingli Wang, Peng Sun, Beng Kang Tay, Ze Xiang Shen, Wenjie Mai and Hong Jin Fan**

P.H. Yang, P. Sun, Prof. W.J. Mai
Department of Physics and Siyuan Laboratory, Jinan University, Guangzhou, Guangdong 510632, China. E-mail: wenjiemai@gmail.com

P.H. Yang, D.L. Chao, C.R. Zhu, Dr. X.H. Xia, Y.Q. Zhang, Prof. Z. X. Shen, Prof. H. J. Fan
School of Physical and Mathematical Sciences, Nanyang Technological University, 637371, Singapore. E-mail: fanhj@ntu.edu.sg

X.L.Wang, Prof. B. K. Tay
School of Electrical and Electronic Engineering, Nanyang Technological University, 639798, Singapore

P.H. Yang and D.L. Chao contributed equally to this work.

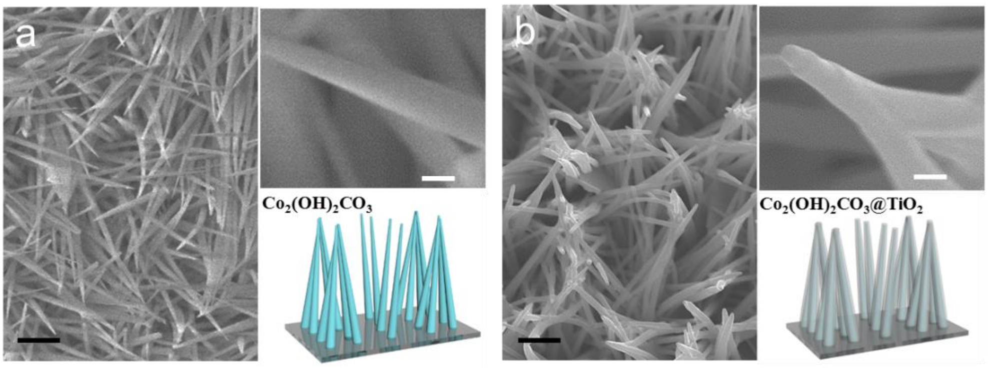

Figure S1. SEM images of $Co_2(OH)_2CO_3$ nanowires precursor and TiO_2 coating. Scale bar: 1 µm in main image and 100 nm in insets.

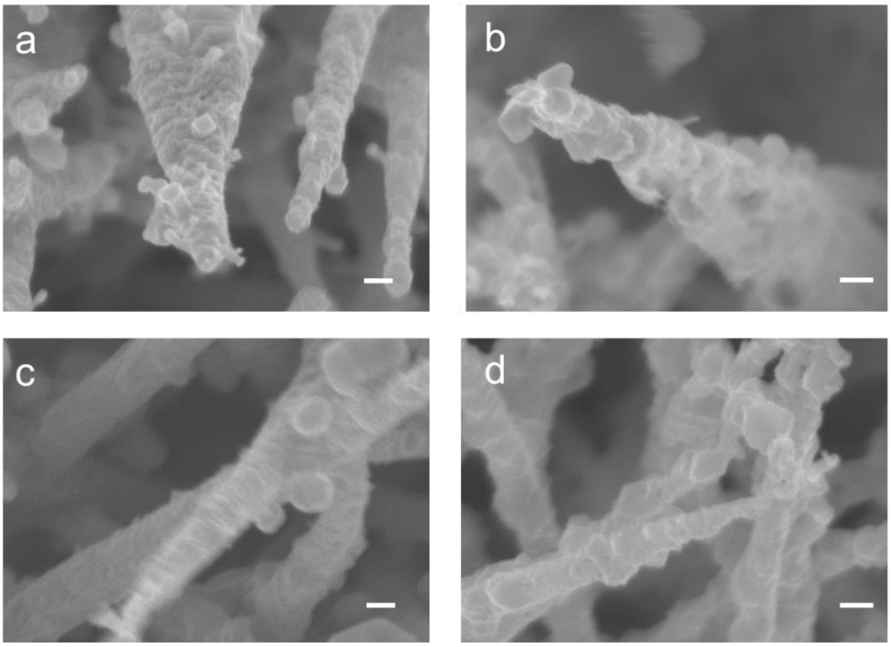

Figure S2. SEM images of (a) TiN-50 and (b) TiN-20 nanostructures on nickel plate, and (c) TiN-50 and (d) TiN-20 nanostructures on stainless steel. Scale bar: 100 nm.

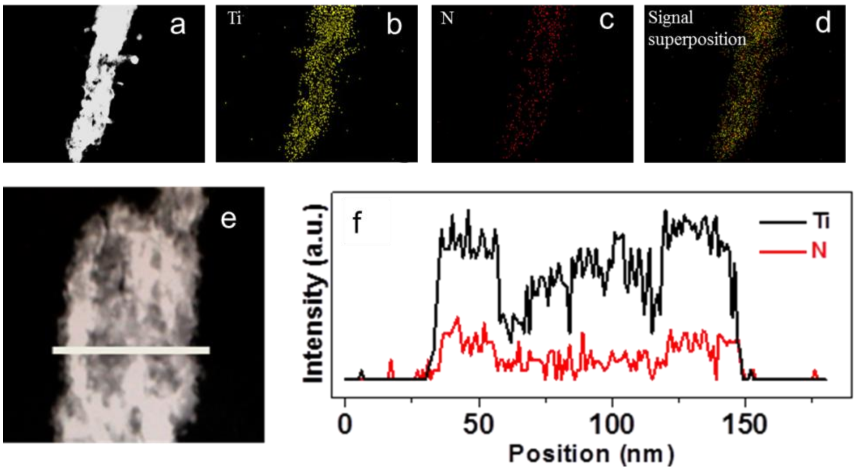

Figure S3. EDS mapping of Ti and N of TiN-20.

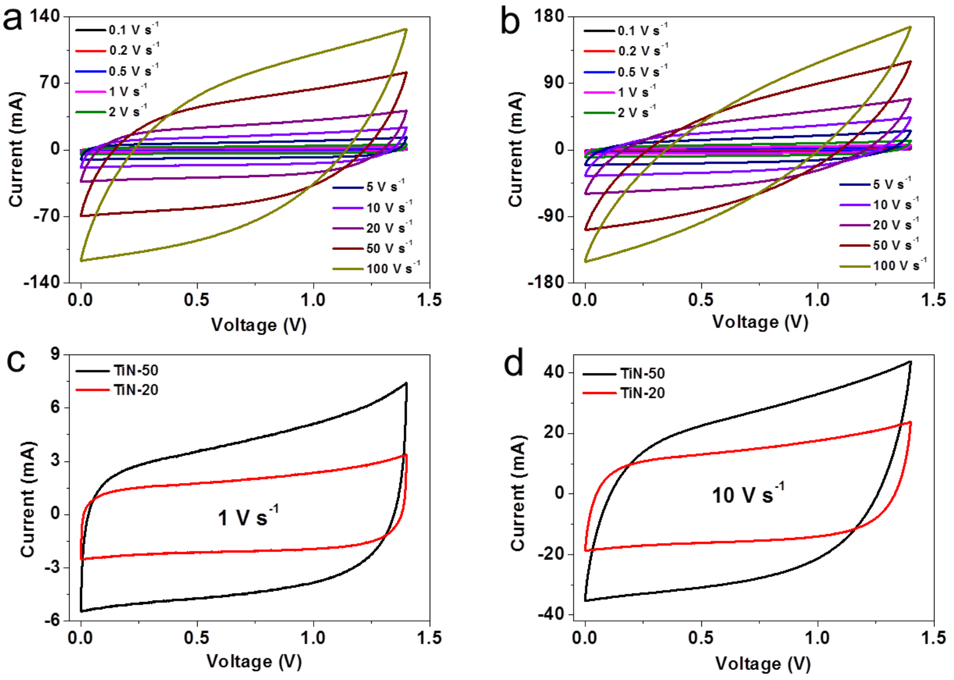

Figure S4. CV curves of (a) TiN-20 and (b) TiN-50 devices. CV curves of TiN-20 and TiN-50 at (c) 1 V s^{-1} and (d) 10 V s^{-1}, respectively.

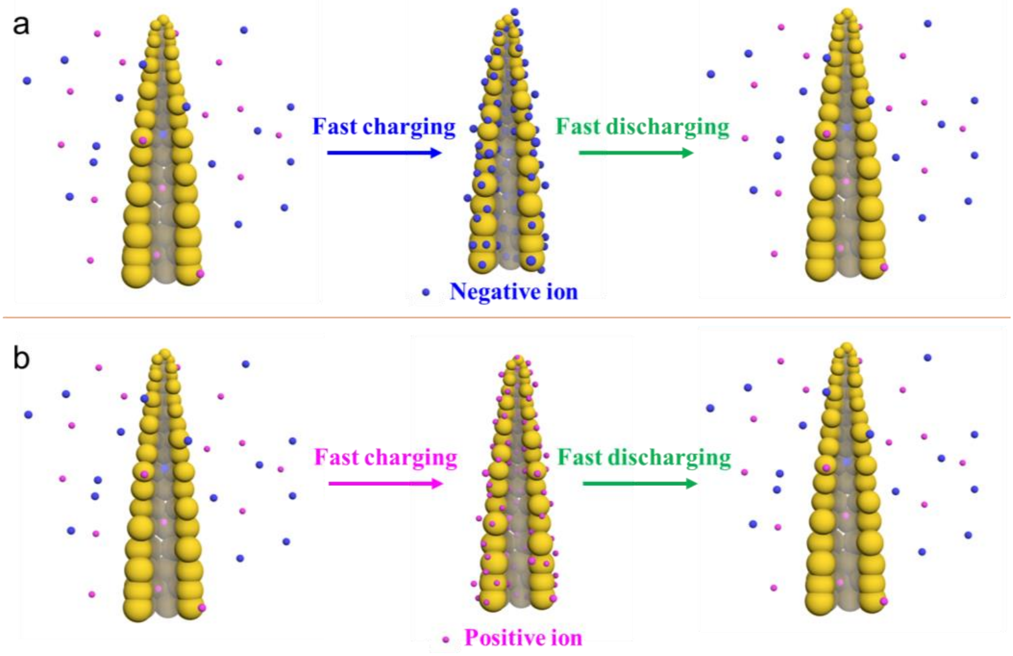

Figure S5 Schematics of the proposed charging and discharging on (a) positive and (b) negative electrodes in the TiN supercapacitors, illustrating the rapid transport of the ions along TiN surface with a short diffusion length.

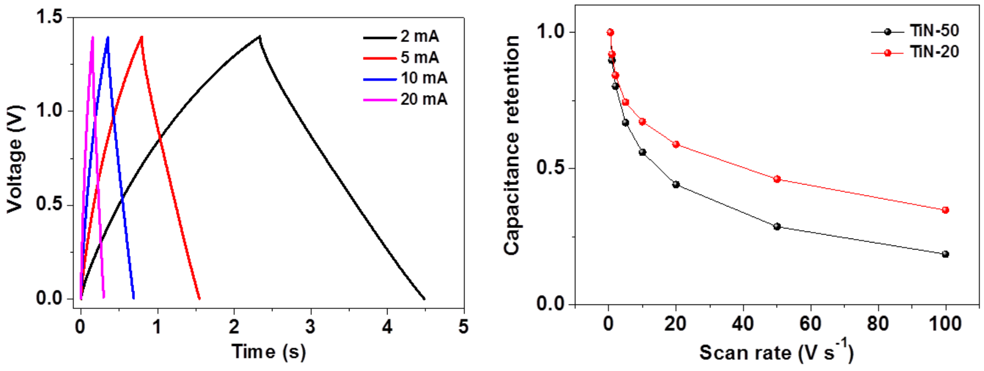

Figure S6. (a) Galvanostatic charge/discharge curves of TiN-20. (b) Capacitance retention of TiN-20 and TiN-50 devices with increasing the scan rate.

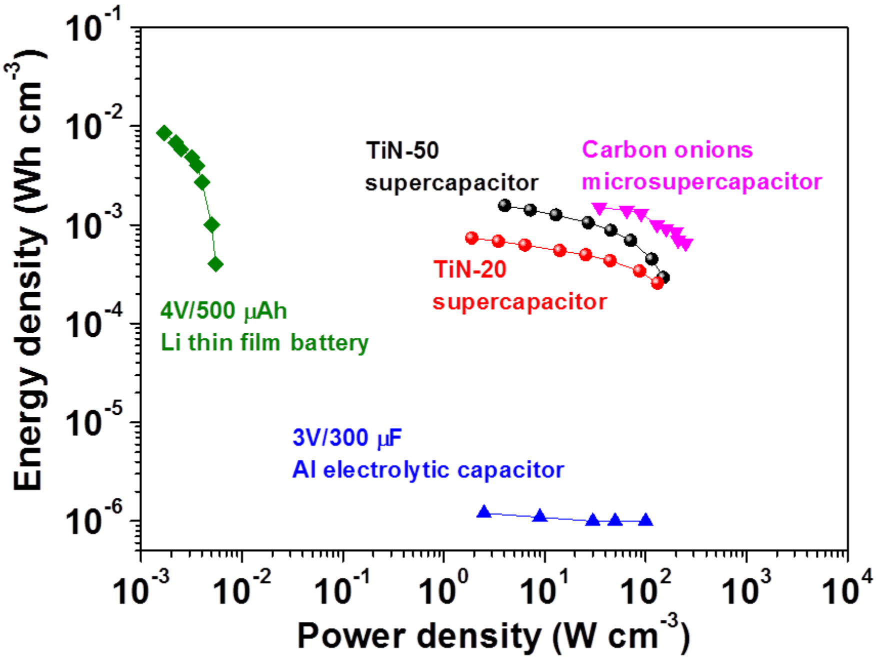

Figure S7. Ragone plots of TiN supercapacitors in comparison to commercial electrolytic capacitors, lithium thin-film batteries and carbon onions micro-supercapacitors.

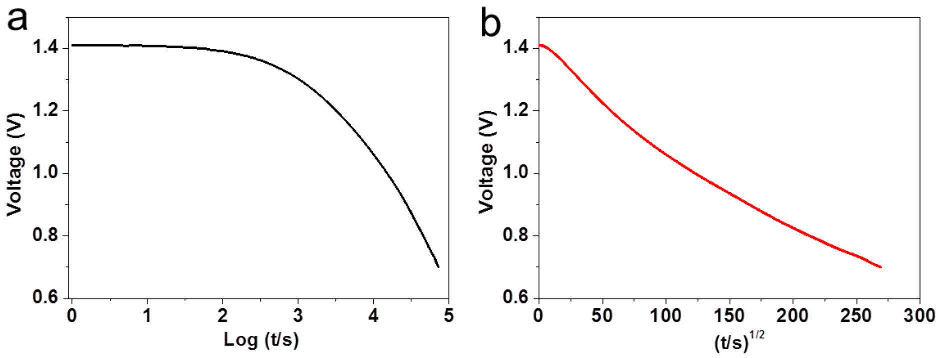

Figure S8. TiN-20 supercapacitor self-discharges in (a) logarithmic time scale and (b) square root time scale. On the basis of previous reports, the self-discharge diagnostic can be performed according to the voltage time dependency. If the voltage drops with an logarithmic law, U(t) *versus* log (t), the mechanisms are controlled by faradic self-discharge from overcharge or from the oxidation-reduction reactions of the impurities. If the voltage drops following a square root function of the time, U(t) *versus* $t^{1/2}$, the mechanisms are controlled by diffusion processes.[1-2] The results show that the self-discharge behaviors of TiN supercapacitor are more likely to be controlled by diffusion processes, just as the Maxwell commercial products (BCAP0007 and BCAP0008, described in Béguin, F.; Frąckowiak, E., *Supercapacitors: Materials, Systems, and Applications*. Wiley-VCH Verlag GmbH & Co. KGaA: Weinheim, 2013, page 380-381).

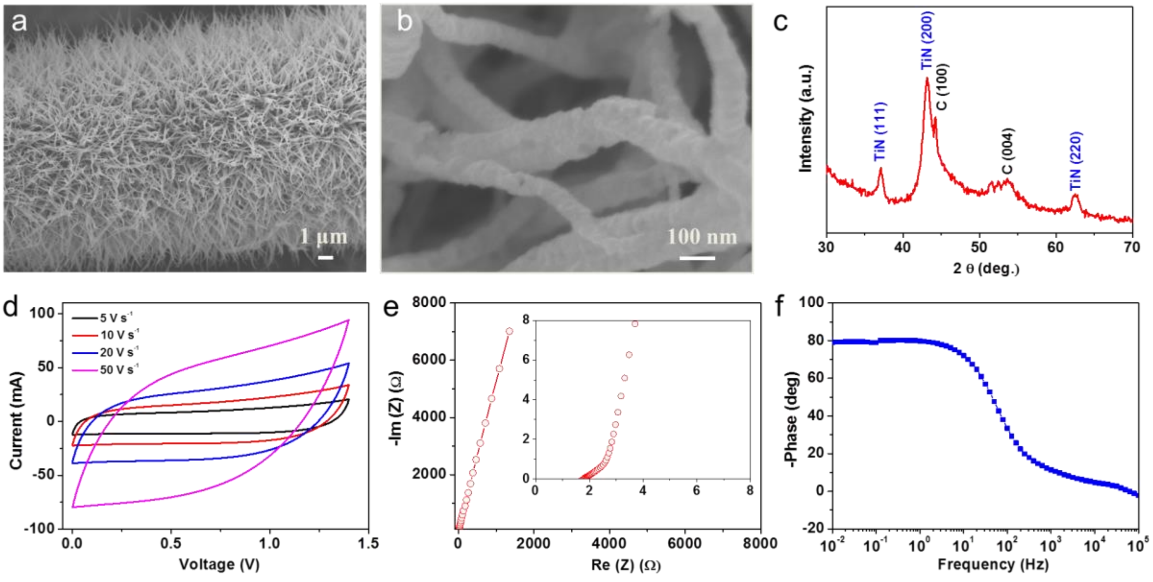

Figure S9. Demonstration of flexible supercapacitors based on TiN nanostructures grown on carbon fabric.

Table S1. Comparison of electrochemical performance of various supercapacitors reported in the literatures.

Reference	Device	Configuration	Electrolyte	Frequency at -45° (Hz)	C_v at 1 Vs^{-1} ($F\ cm^{-3}$)	E ($mWh\ cm^{-3}$)	P ($W\ cm^{-3}$)
**[3]**	Nanochanneled PDDA-Graphene	Micro	H_2SO_4/PVA	30	~80	2–6.7	0.1–500
**[4]**	laser-scribed graphene	Micro	H_2SO_4/PVA	53	~2.3	0.2–0.4	0.6–60
**[5]**	methane plasma graphene	Micro	H_2SO_4/PVA	3579	~10	0.14–2.5	0.1–495
**[6]**	Onion-like carbon	Micro	1 mol L^{-1} Et_4NBF_4/PC	38	1.3	0.65–1.5	35–-250
**[7]**	MnO_2@carbon fiber	Micro	H_3PO_4/PVAs	45	~0.8	1.2–2.2	0.008–0.4
**[8]**	Graphene-Bridged V_2O_3/VO_x	Conventional	1 mol L^{-1} H_2SO_4	114	N/A	N/A	N/A
**[9]**	PEDOT	Micro	1 mol L^{-1} H_2SO_4	400	~13	2.2	3.5
**[10]**	PEDOT/MWNT	Micro	H_2SO_4/PVA	12.5	145	0.3–1.4	3–40
**[11]**	MnO_x/Au	Micro	H_2SO_4/PVA	200	32.8	1–1.75	0.08–3.44
**Our work**	TiN-20	Conventional	1 mol L^{-1} $LiClO_4$/PC	80	10.0	0.26–0.7	1.9–132
**Our work**	TiN-50	Conventional	1 mol L^{-1} $LiClO_4$/PC	40	20.7	0.3–1.5	4–150

References

[1] F. Béguin, E. Frąckowiak, *Supercapacitors: Materials, Systems, and Applications*. Wiley-VCH Verlag GmbH & Co. KGaA: Weinheim, 2013.

[2] B. E. Conway, *Electrochemical Supercapacitors: Scientific Fundamentals and Technological Applications*. Kluwer Academic / Plenum: New York, 1999.

[3] J. Chang, S. Adhikari, T. H. Lee, B. Li, F. Yao, D. T. Pham, V. T. Le, Y. H. Lee, *Adv. Energy Mater.* **2015,** 5, DOI: 10.1002/aenm.201500003.

[4] M. F. El-Kady, R. B. Kaner, *Nat. Commun.* **2013,** 4, 1475.

[5] Z. S. Wu, K. Parvez, X. Feng, K. Müllen, *Nat. Commun.* **2013,** 4, 2487.

[6] D. Pech, M. Brunet, H. Durou, P. Huang, V. Mochalin, Y. Gogotsi, P.-L. Taberna, P. Simon, *Nat. Nanotechnol.* **2010,** 5, 651.

[7] X. Xiao, T. Li, P. Yang, Y. Gao, H. Jin, W. Ni, W. Zhan, X. Zhang, Y. Cao, J. Zhong, L. Gong, W.-C. Yen, W. Mai, J. Chen, K. Huo, Y.-L. Chueh, Z. L. Wang, J. Zhou, *ACS Nano* **2012,** 6, 9200.

[8] X. Pan, G. Ren, M. N. F. Hoque, S. Bayne, K. Zhu, Z. Fan, *Adv. Mater. Interfaces* **2014,** 1, DOI: 10.1002/admi.201400398.

[9] N. Kurra, M. K. Hota, H. N. Alshareef, *Nano Energy* **2015,** 13, 500.

[10] J. A. Lee, M. K. Shin, S. H. Kim, H. U. Cho, G. M. Spinks, G. G. Wallace, M. D. Lima, X. Lepró, M. E. Kozlov, R. H. Baughman, S. J. Kim, *Nat. Commun.* **2013,** 4, 1970.

[11] W. Si, C. Yan, Y. Chen, S. Oswald, L. Han, O. G. Schmidt, *Energy Environ. Sci.* **2013,** 6, 3218.
